# *SeqCNV:* a novel method for identification of copy number variations in targeted next-generation sequencing data

**DOI:** 10.1186/s12859-017-1566-3

**Published:** 2017-03-03

**Authors:** Yong Chen, Li Zhao, Yi Wang, Ming Cao, Violet Gelowani, Mingchu Xu, Smriti A. Agrawal, Yumei Li, Stephen P. Daiger, Richard Gibbs, Fei Wang, Rui Chen

**Affiliations:** 1 Shanghai Key Lab of Intelligent Information Processing, Shanghai, China; 2 School of Computer Science and Technology, Fudan University, Shanghai, China; 3 Structural and Computational Biology & Molecular Biophysics Graduate Program, Baylor College of Medicine, Houston, TX USA; 4 Human Genome Sequencing Center, Baylor College of Medicine, Houston, TX USA; 5 School of Life Sciences, Fudan University, Shanghai, China; 6 University of Texas Health Science Center, Houston, TX USA; 7 Department of Molecular and Human Genetics, Baylor College of Medicine, Houston, TX USA; 8 Department of Ophthalmology and Visual Sciences, University of Texas Health Science Center, Houston, TX USA

**Keywords:** Next-generation sequencing, Copy number variation, Maximum penalized likelihood estimation

## Abstract

**Background:**

Targeted next-generation sequencing (NGS) has been widely used as a cost-effective way to identify the genetic basis of human disorders. Copy number variations (CNVs) contribute significantly to human genomic variability, some of which can lead to disease. However, effective detection of CNVs from targeted capture sequencing data remains challenging.

**Results:**

Here we present SeqCNV, a novel CNV calling method designed to use capture NGS data. SeqCNV extracts the read depth information and utilizes the maximum penalized likelihood estimation (MPLE) model to identify the copy number ratio and CNV boundary. We applied SeqCNV to both bacterial artificial clone (BAC) and human patient NGS data to identify CNVs. These CNVs were validated by array comparative genomic hybridization (aCGH).

**Conclusions:**

SeqCNV is able to robustly identify CNVs of different size using capture NGS data. Compared with other CNV-calling methods, SeqCNV shows a significant improvement in both sensitivity and specificity.

**Electronic supplementary material:**

The online version of this article (doi:10.1186/s12859-017-1566-3) contains supplementary material, which is available to authorized users.

## Background

The development of Next-Generation Sequencing (NGS) technologies has enabled the generation of large-scale sequence datasets. The ability to identify and characterize genomic variants and mutations from large numbers of individuals has become feasible, driving advances in our understanding of genetic diseases. Due to the cost and the complexity of analyzing whole genome sequence data, targeted capture sequencing has become the predominant approach for genetic diagnostic purposes. Targeted capture sequencing yields significantly greater depth of coverage, providing increased quality and fidelity at a decreased cost compared with whole genome sequencing [1–4]. However, a major limitation of capture NGS is that only single nucleotide variants (SNVs) and small insertions and deletions (Indels) can be identified, while large duplication and deletions are ignored in most cases because copy number variation (CNV) identification from targeted NGS data is less reliable.

CNVs are large genomic DNA segments (≥1 kb) with variable copy number among individuals [5]. A substantial proportion of the human genome is copy number variable and more than a thousand CNV regions with a frequency of greater than 1% have been identified in the genome [6]. CNVs encompassing genes can potentially alter gene dosage, disrupt genes or perturb their expression levels [7], and are known to contribute to a number of disorders [8–15]. Additionally, CNVs have played a pivotal role in evolutionary [16–18] and population genetics analysis [19, 20].

Traditional methods for CNV identification include array comparative genomic hybridization (aCGH) [21] and SNP array technologies. In recent years, NGS has provided alternative approaches to assay CNVs [22–25]. There are two primary strategies for CNV detection using NGS data: paired-end mapping (PEM) and depth of coverage (DOC). In the PEM-based methods, both paired ends of a sequenced fragment are aligned against the reference genome, and discordantly mapped paired reads whose distances are significantly deviated from the mean insert size of fragments are predicted to possess alternations in copy number [26]. PEM-based methods are not suitable for targeted NGS as they are limited by the read length when finding large copy number gains. More importantly, they require that paired reads cross the junction. Since CNV boundaries are more likely to be located in introns or intergenic regions that are far from the targeted regions, many CNVs will be completely missed by PEM-based approaches that use targeted capture sequencing data. Another type of CNV calling methods is based on DOC windows [27]. The underlying approach is to compare the differences of DOC in particular genomic regions between case and control samples [28, 29]. Unlike the PEM-based methods that are limited by the insert size and can only detect smaller CNVs, the DOC-based methods can, in theory, detects arbitrarily large insertions. Furthermore, DOC can be effectively used with paired-end, single-end, and mixed read data. However, due to large variation of the capture NGS data, DOC-based methods usually result in significant false positives [30].

Currently, several methods have been developed to identify CNVs from capture NGS data, including CoNIFER [31], CNVnator [32], CNVer [33] and XHMM [34]. CoNIFER exploits singular value decomposition (SVD), which aims to eliminate capture biases among sample batches. XHMM is based on principal component analysis (PCA) normalization and hidden Markov model (HMM). Reliance on SVD and PCA limit the ability of CoNIFER and XHMM to perform CNV calling with a large number of samples. CNVnator and CNVer are both DOC-based methods. CNVer supplements the DOC with paired-end mapping information, where mate pairs mapping discordantly to the reference indicate the presence of variation [33]. Both of them can identify a large number of CNVs but are not effective in detecting small-size CNVs [35, 36].

To address the limitations of current methods in detecting CNVs using target capture NGS data, we developed a robust statistical method called SeqCNV, which uses maximum penalized likelihood estimation (MPLE) to evaluate the CNV boundary and the copy number ratio. Given the variation of the sequencing depths of the case and control samples, normalization was performed using the total reads number for each chromosome in the likelihood model. A novel segmentation algorithm was developed which enabled the detection of CNVs with different lengths. We also present an assessment of its sensitivity, specificity and limitations using targeted sequencing data from both bacterial artificial clone (BAC) and human patients.

## Methods

### Statistical modeling and algorithm

**Fig. 1:**
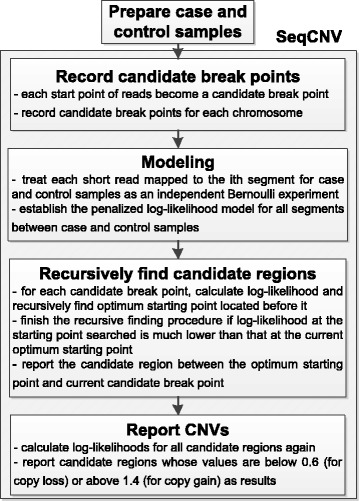
Workflow of SeqCNV. A dynamic programming procedure is included in the step “Recursively find candidate regions”. It aims to quickly break iteration to find candidate regions that are most likely to be CNV events, thereby saving much time for whole algorithm running

SeqCNV is a DOC-based method to identify CNVs from target capture NGS data. The workflow of SeqCNV includes four steps as shown in Fig. 1. First, with provided case and control samples, SeqCNV considers the starting point of each read as a candidate break point (CBP). Next, SeqCNV establishes the penalized log-likelihood model for all segments between case and control samples. For each CBP, SeqCNV calculates the likelihood and recursively finds the optimum starting point located upstream of it. Once the optimum starting point is determined, the region between it and the CBP is considered as a candidate CNV region. Finally, SeqCNV reports candidate regions whose likelihood values are below 0.6 (for copy loss) or above 1.4 (for copy gain). In the segmentation stage, each chromosome can be partitioned into *n* segments with *n*-1 breakpoints. When case and control reads are mixed together and given a read that is successfully mapped to the *i*th segment, we assume the probability that the read is from the case sample (*p*_*i*_) is homogeneous across the entire targeted region. Thus, the control is diploid and the *p*_*i*_ is normalized to the diploid genome, which is the relative probability. Therefore, the probability that the read is from the control sample is 1-*p*_*i*_. Thus, each short read mapped to the *i*th segment is an independent Bernoulli experiment with two outcomes: being a case read or a control read. *t*_*i*_ and *c*_*i*_ are denoted as the number of reads mapped to the *i*th segment in case and control samples, respectively. The log-likelihood *L* of the model is as follows:

1 $$ L={\displaystyle \sum_i{t}_i \ln {p}_i+{c}_i \ln \left(1-{p}_i\right)}. $$

Each segment has two parameters to be determined: the left boundary position and the copy number ratio, with the exception of the first segment, which only required the copy number ratio. Thus, the model contains 2*n*-1 parameters.

The optimization goal is to minimize the number of segments while keeping its fitness to the data. This task can be achieved by several criteria, such as *p*-value-based statistical testing, Akaike’s information criterion (AIC) [37] and Bayesian information criterion (BIC) [38]. All these criteria can be viewed as particular instances of MPLE, which attempts to maximize the following penalized likelihood (PL):

2 $$ PL={\displaystyle \sum_i\left({t}_i \ln {p}_i+{c}_i \ln \left(1-{p}_i\right)\right)-\lambda \left(2 n-1\right)}. $$

In this equation, *λ* is the penalization factor, as $\lambda =\frac{1}{2}{\chi}_p^2$ for the *p*-value-based criterion, *λ* = 1 for AIC and $\lambda =\frac{1}{2} \ln N$ for BIC, where *N* is the total number of reads in the targeted genome. We recommended BIC owing to its robust statistical properties such as minimum description length [39, 40].

To find the MPLE, we proposed a dynamic programming procedure. Suppose there are *M* CBPs. Let *s* (*j*, *i*) be the log-likelihood of the segment that starts from the *j*th CBP and ends at the *i*th CBP.

3 $$ s\left( j, i\right)= t \ln \frac{t}{t+ c}+ c \ln \left(\frac{c}{t+ c}\right), $$

where *t (c)* is the number of reads mapped in the segment in the case (control). Denote *b*(*i*) as the maximum penalized log-likelihood of the chromosome started at the beginning and ended at the *i*th CBP. Denote *B* (*i*) as the best starting CBP of the segment that ended at the *i*th CBP. The recursion formula is as follows:

4 $$ b(i)=\left\{\begin{array}{l}0\kern0.84em  i=0\\ {}\kern.01em  s\left(1,1\right)-\lambda \kern0.36em  i=1\\ {} \max \left( s\left( j, i\right)+ b\left( j-1\right)-2\lambda, 0< j\le i\right)\kern0.48em 1< i\le M,\end{array}\right. $$

5 $$ B( i)=\Big\{\begin{array}{l}0\kern0.6em  i=1\\ {}{ \max}_j\left( s\left( j, i\right)+ b\right( j-1\left)-2\lambda, 0< j\le i\right)\kern0.6em 1< i\le M\end{array}\operatorname{} $$

The recursion formula has a computational complexity of *O*(*n* ^2^). For implementing this dynamic procedure, double loops are necessary in the coded program. The outer loop is for each *i* from 1 to M, traversing M CBPs. The inner loop is for *j*, decreasing from *i* to 1, searching the optimum starting point from the ending point *i*. To speed up, the inner loop will stop if the penalized log-likelihood at *j* is much lower than that at the current optimum starting point, *s* (*j*, *i*) + *b* (j-1)-2*λ* < *b*(i)_j_-*const*, where in our experiment, *const* = 5*λ*.

If the dataset contains less than 1 M shorts reads, each read can be set as a CBP and the best partition can be found within several hours with read level resolution. For larger datasets, first, CBPs can be selected instead of treating each read as a CBP as described [28]. To identify the CBP set, the dynamic programming algorithm described in formula (4) could also be utilized with lower penalty, for instance, *λ*_= 1.92_, which is equivalent to a *p*-value of 0.05 threshold. After CBPs being determined, the formula (4) will be run again with bigger *λ* to solve the optimization problem of formula (2).

### Simulation dataset preparation

To evaluate the detection power and the false positive rate (FPR) of SeqCNV with different lengths of CNVs, we generated sequencing reads with the starting position based on the NimbleGen CCDS design file on chromosome 1, which includes 8315 targets with an average length of 168 base pairs. Detection power is defined as how many simulated one-copy gains or losses are covered by the segments that have close ratios. FPR is defined as how many segments whose ratios indicate more than one copy change actually do not overlap with the simulated ones. We assumed that the number of reads for each target followed a Poisson distribution with as the product of the affinity and length, and coordinated within the range being sampled. For the rest of the chromosome, off-target reads were assumed to be uniformly distributed and randomly sampled.

### BAC spike-in experiment

DNA of nine non-overlapping BAC clones were spiked into control human genomic DNA (Additional file 1). The spike-in sample was used to mimic copy number gains in nine targeted regions.

### Retinitis pigmentosa (RP) patient data analysis

RP is an inherited form of retinal degenerative disease causing progressive vision loss. Autosomal dominant RP (adRP) can be caused by loss of a single copy of *PRPF31* gene on chromosome 19. To test the performance of our method, we applied SeqCNV on five adRP patients who were known to carry *PRPF31* deletions. Patient DNA was extracted from peripheral blood using standard techniques.

### Targeted panel design and sequencing data analysis

A custom capture panel was designed using AgilentSureSelect (Agilent Technologies, CA) targeting 18 genes (*RPGR*, *TULP1*, *CABP4*, *RGR*, *MYO7A*, *PRPF31*, *ABCA4*, *USH2A*, *CNGB1*, *SAG*, *RPGRIP1*, *LCA5*, *CNGA1*, *MERTK*, *CLRN1*, *TTC8*, *CC2D2A*, and *PDE6A*). All normal control samples, BAC spike-in samples, and adRP patient samples were sequenced using the same panel in the same batch. Resultant DNA was bar-coded, prepared, and shotgun sequenced. Reads were aligned to Human Reference Genome hg19 using Burrows Wheeler Aligner (BWA) [41]. Recalibration and realignment were performed using The Genome Analysis Toolkit (GATK) [42]. Samtools [43] was used to sort and index the resultant BAM files. Quality control analysis showed that 97% and 81% of the target area were covered with > 10× and > 40×, respectively.

### aCGH validation

To validate the CNVs identified from NGS, we performed aCGH experiments on the patients with adRP. A customized aCGH platform targeting the same 18 genes including the *PRPF31* (MIM: 606419) was designed using Agilent Suredesign (https://earray.chem.agilent.com/suredesign). The probes used are available upon request. The aCGH experiments were performed as per the manufacturer’s instructions and were analyzed using Agilent Genomic Workbench.

## Results

### Simulated results

**Fig. 2:**
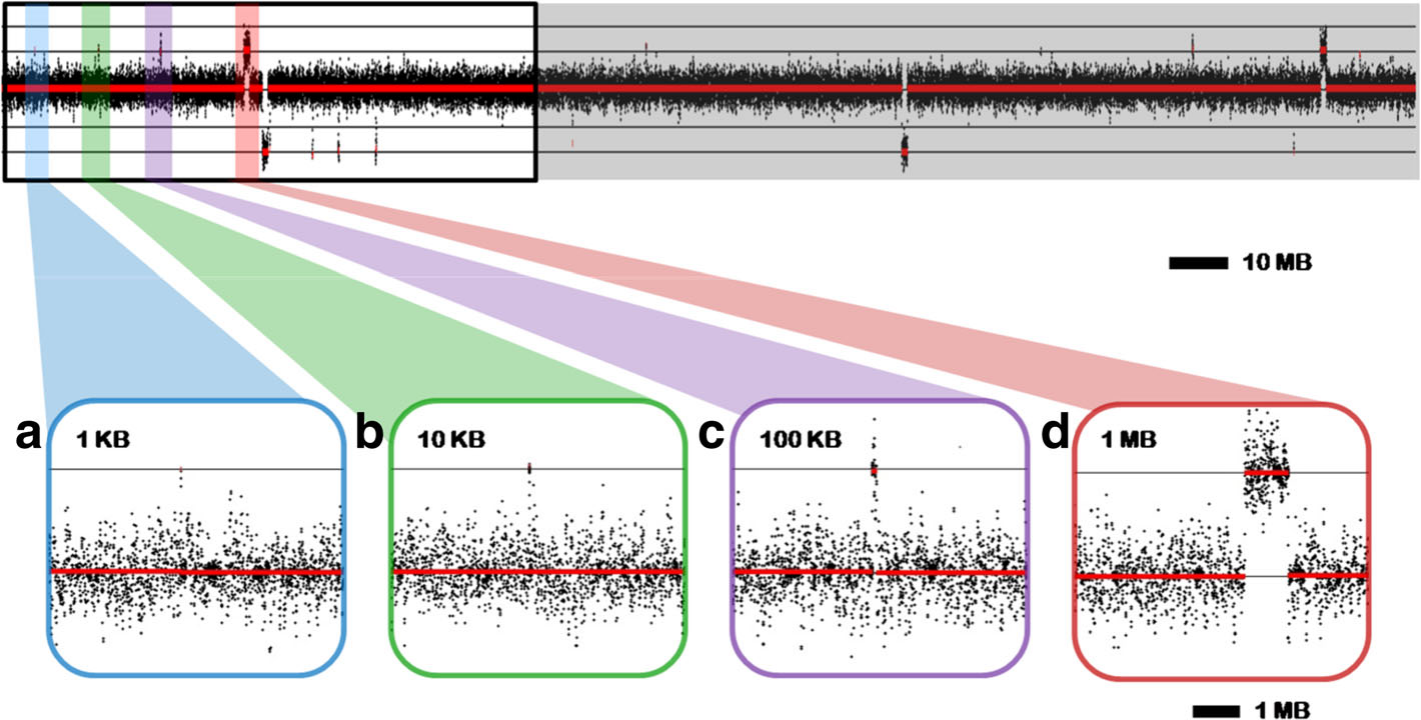
An example of simulated CNV data on chromosome 1. The data set, computationally simulated, includes two deletions and two duplications at each of four lengths. *Black* dots represent read density over 500 bp fixed windows along the entire chromosome. The *red* bands indicate the results of SeqCNV analysis. Horizontal lines mark significant points of deletion or gain

We simulated both case and control data and applied SeqCNV to obtain the segmentations. We performed this process for 100 rounds. In each run, we randomly generated four copy changes including two gains and two losses at different sizes of 1 MB, 100 KB, 10 KB and 1 KB, containing at least one captured exon. For each of these 16 changes per sample, we mimicked a single copy number gain or loss by increasing or decreasing the number of case reads by 50% relative to the control, respectively. As shown in Fig. 2, simulated copy number changes of different sizes can be detected.

As shown in Table 1, SeqCNV is sensitive to gains of 1 KB and losses of 1 KB. Sensitivity was calculated as the ratio of correctly detected CNV regions to the total number of simulated CNV regions. A detected region was considered as a true positive only if SeqCNV determined a copy gain ratio no greater than 1.4, or loss ratio no less than 0.6 (complying with the ideal ratios of 1.5 for gain and 0.5 for loss). Additionally, the overlap of copy loss detections and simulated regions was required to exceed 50%. Due to the detection difficulty of copy number gains, any overlap of simulated regions was deemed sufficient for copy gain detections [44]. It is noted that high sensitivity is associated with 1 KB regions, which is indicative of an ability to detect a single exon copy number gain or loss.

**Table 1:** Summary of 100 runs of SeqCNV on simulation data. Boundary (Start/End) is the average distance to the nearest starting (ending) point of the detected variants

Type	One copy gain				One copy loss			
Resolution	1 MB	100 KB	10 KB	1 KB	1 MB	100 KB	10 KB	1 KB
Sensitivity	99.50%	99.00%	75.00%	67.80%	99.00%	96.50%	91.00%	66.80%
Boundary (Start)	1.69 KB	1.37 KB	0.73 KB	0.29 KB	0.71 KB	0.64 KB	0.49 KB	0.18 KB
Boundary (End)	1.28 KB	1.01 KB	0.91 KB	0.32 KB	0.91 KB	0.71 KB	0.72 KB	0.21 KB
False Positive Rate	0%	0%	0%	0%	0%	0%	0%	3.74%

### Comparison of performance on BAC spike-in data

One of the challenges in evaluating the performance of CNV callers is the lack of a gold standard. To address this issue, we spiked in equal molar BAC DNA for nine regions into control genomic DNA to mimic copy number gains (see Additional file 1). Using this dataset, the performance of SeqCNV was compared with previous published tools in detecting CNVs from targeted NGS data.

**Fig. 3:**
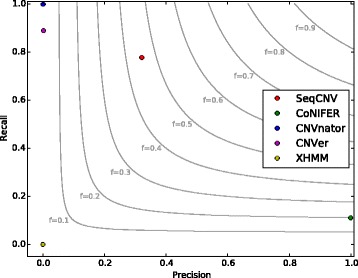
Precision-Recall Contours for five CNV methods on spike-in data. Light grey contours represent F-measure levels (harmonic mean of precision and recall). SeqCNV achieved the highest F-measure value

The results are shown in Table 2. CNVnator and CNVer reported a large number of candidate CNVs, most of which were long regions whose lengths surpassed 1Mbp and turned out to be false positives. CoNIFER identified two CNVs, and both were located in the designed CNV regions, indicating high specificity but relatively low sensitivity. XHMM predicted a few CNV events, but none of them overlapped with the spiked in CNV regions. Precision is calculated as the ratio between the number of correctly detected events (the intersection between the tool calls and the known calls) and the total number of events detected by a tool [45]. The recall is calculated as the ratio between the number of correctly detected events and the total number of events in the validation set [45]. From comparative analysis of the methods, SeqCNV showed a more balanced recall and precision (Fig. 3). CNVnator is the best for recall, followed by CNVer, SeqCNV, CoNIFER and XHMM. The high recall of CNVnator is attributed to its large number of called long CNV regions, which also leads to the low precision. CoNIFER is superior in terms of precision, followed by SeqCNV, CNVer, CNVnator and XHMM. However, the recall of CoNIFER is very low since only two CNVs were called. In comparison, SeqCNV achieved high precision and moderate recall. Overall, from the results on the BAC spike-in data, SeqCNV showed the best trade-off between high precision and recall comparing with the other four methods.

**Table 2:** Number of predicted CNV events and correctly detected events for each method on BAC spike-in data

	SeqCNV	CoNIFER	CNVnator	CNVer	XHMM
Number of Predicted CNV events	53	2	8032	4487	2
Number of Correctly Detected events	7	2	9	8	0

### Comparison of performance on adRP patient data

**Fig. 4:**
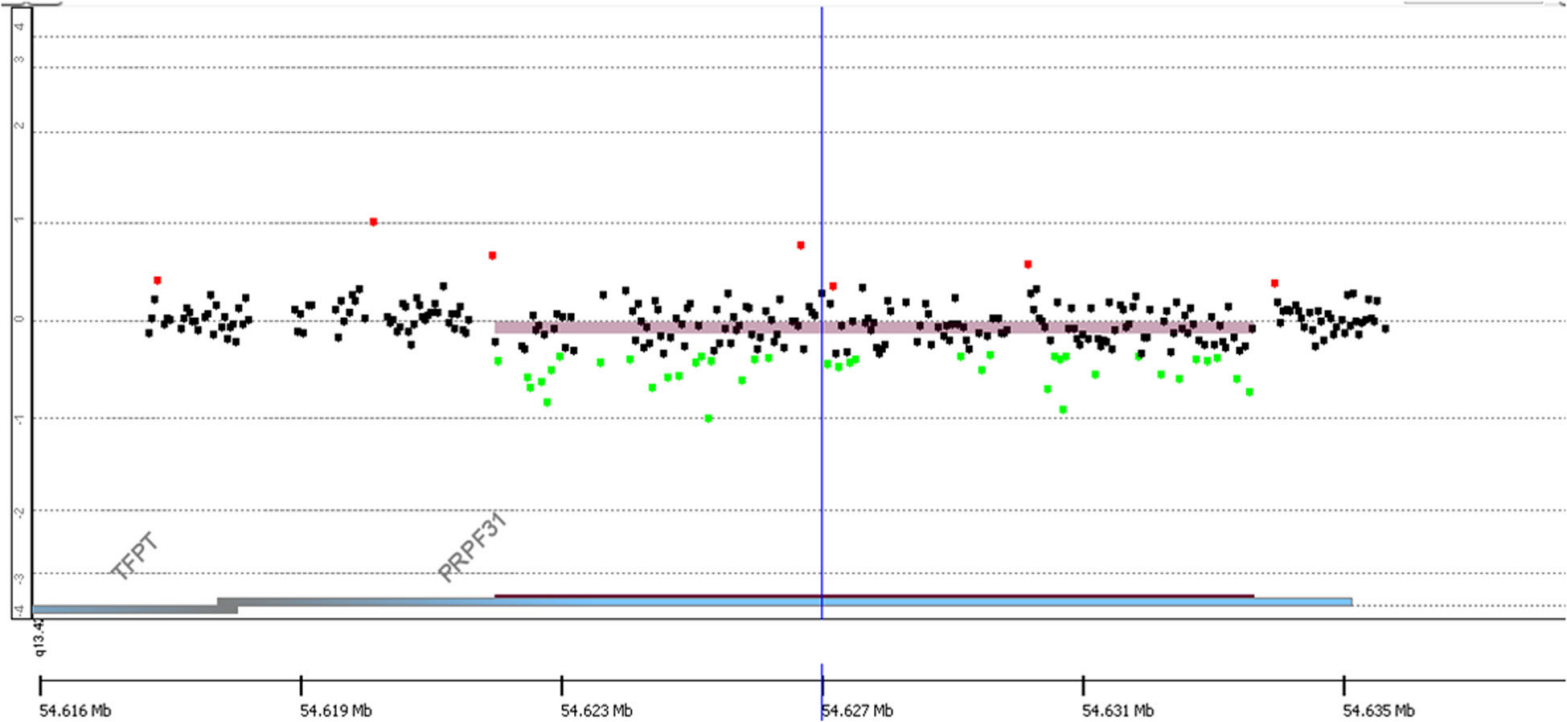
aCGH validation of copy number deletion in *PRPF31* gene for sample UTAD082. The shaded area indicates the CNV area with loss of one copy of the genomic segment. Validation results for other 4 samples can be found in Additional file **1**. Five samples share different deletion sizes, ranging from several exons to entire genomic region of *PRPF31*

**Fig. 5:**
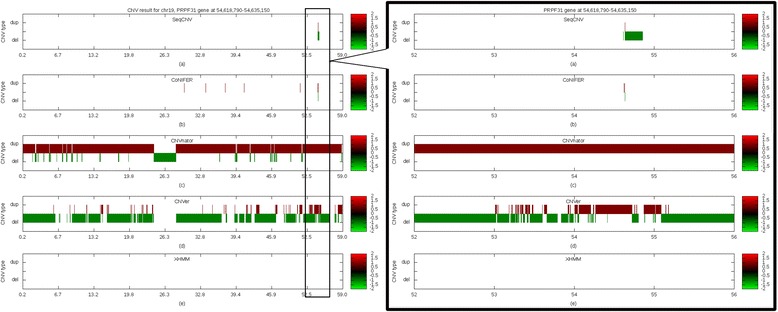
CNV result for five methods on adRP patient data. **a** SeqCNV **b** CoNIFER **c** CNVnator **d** CNVer **e** XHMM. X-axis represents the genomic position for chromosome chr19. *PRPF31* gene is located at chr19:54,618,790–54,635,150. Both SeqCNV and CoNIFER identified the *PRPF31* copy number deletion

We collected targeted NGS data from five patients with adRP. Normal control samples were sequenced in the same batch. Each of these case samples contained a copy number loss in *PRPF31*, which was validated by aCGH (Fig. 4, Additional file 2). Considering the possibly of a non-even read distribution, we selected four samples without a copy change in *PRPF31* and merged these samples with the control set. With five patient samples and the merged control samples, we ran all the CNV tools and the obtained results shown in Table 3 and Fig. 5. For CoNIFER and XHMM, we added an extra 46 samples without copy change in *PRPF31* as controls due the requirement of SVD and PCA methods. The criteria of control sample selection are described in the Additional file 3. As shown in Fig. 5, both SeqCNV and CoNIFER identified deletions in *PRPF31* on chromosome 19. However, CoNIFER resulted in a larger FPR. Consistent with our previous BAC spike-in experiment, both CNVnator and CNVer reported large number of CNVs, almost covering the entire chromosome. XHMM did not give any positive results. Therefore, SeqCNV outperformed the other four methods in this comparison.

**Table 3:** Number of predicted CNV events and correctly detected events for each method on adRP patient data

Sample ID	CNV	SeqCNV	CoNIFER	CNVer	CNVnator	XHMM
UTAD034	*PRPF31* entire gene deletion	Y	Y	N	N	Na
UTAD069	*PRPF31* exon 4–8 deletion	Y	Y	N	N	Na
UTAD082	*PRPF31* exon 4–13 deletion	Y	Na	N	N	Na
UTAD411	*PRPF31* entire gene deletion	Y	Y	N	N	Na
UTAD611	*PRPF31* exon 1–11 deletion	Y	Y	N	N	Na

Each element in the table indicates that whether copy number deletion for genomic region of gene *PRPF31* in that sample is identified by the CNV method or not. ‘Na’ element indicates the method did not report any CNV for the sample. As we can see, SeqCNV really identified all the copy number deletion for all the 5 samples

### Execution time estimation

Since efficiency is an important factor to consider in evaluating the performance of the tools, we tested SeqCNV with other four methods on the BAC spike-in data, adRP human patients data and whole-exome sequencing (WES) data obtained from the 1000 Genome projects (ftp://ftp.1000genomes.ebi.ac.uk/) [46, 47]. As shown in Additional file 4, SeqCNV required 4–5 min to analyze the BAC spike-in and adRP patient data, and about 17 min to process chromosome 1 data from WES. Compared with other tools, SeqCNV was the most efficient in performing the processing and analysis of the datasets.

## Discussion

SeqCNV offers many advantages over previously used methods. It does not require a large number of samples to run and is able to detect CNVs of different sizes by treating the left boundary of each reads as a CBP. As shown in both BAC spike-in data and adRP human patient data, SeqCNV exhibited the highest accuracy compared with other methods.

SeqCNV can also be effectively used to analyze patient data for other regions potentially causing additional genetic diseases. Besides BAC spike-in and RP patient data, we also tested SeqCNV on WES samples from the 1000 genome project (ftp://ftp.1000genomes.ebi.ac.uk/). Similar to the methods used for analysis of the adRP patient data, we considered multiple factors such as DNA quality, DNA extraction protocol and the possibly non-even reads distribution and randomly selected three samples to pool together as a control, and randomly selected one sample as case. We validated our results with the CNVs previously reported by Conrad et al. [46, 47] in the WES samples. The results are shown in Additional file 5. Overall, SeqCNV was able to identify the known CNVs with a good recall rate (55%) and low false positive rate (~10%).

The study of CNVs in human disease is a rapidly evolving field. CNVs can result in gene dosage changes and give rise to a substantial amount of human phenotypic variability. It also has been shown that CNVs play an essential role in cancer [46, 47]. However, currently investigating CNVs for human disease is still largely overlooked due to technical issues, such as the limited accuracy of CNV detection methods from NGS data. Therefore, it is important to increase the sensitivity of detection while controlling the false positive rate with statistical tools. We recognize that although SeqCNV demonstrated the best trade-off between precision and recall compared to other approaches in our tests, it does still result in a significant number of false positives, especially when the sequencing quality is not reliable. In addition, the performance of CNV tools based on targeted capture sequencing would be limited by the capture design, although it is very efficient in detecting CNVs in known pathogenic genes. Contrarily, whole-genome sequencing may be more effective in detecting CNVs in novel regions. However, because of factors such as cost, it is not so widely used in clinical applications.

As shown in the simulation results, the length of the CNV will affect the sensitivity of detection. It is easier to detect larger CNVs while smaller size CNVs are sometimes indistinguishable from the background. One possible solution is to increase the sequencing depth, which will improve the statistical power of SeqCNV. In addition, using matched case and control files can help reduce the number of false positives resulting from sequencing bias.

Based on our comparative analysis, we observed that CNVer and CNVnator predicted a large number of CNVs. Both methods shared good recall but high FPR. Although SeqCNV requires matched control samples to perform the analysis, we can also derive a control sample by pooling other samples together, which will still serve as an effective control. As shown in the *PRPF31* deletion analysis of adRP patients, we combined four normal samples that were sequenced in the same batch as the control. Furthermore, since sequencing quality can be affected by multiple factors, including DNA quality, and DNA extraction protocol, it is recommended that users select samples with good quality to pool together as controls. For example, users can calculate the evenness scores [48] representing the uniformity of sequencing and samples with highest evenness scores can be pooled as controls.

## Conclusions

In this study, we devised a novel method, SeqCNV, based on the MPLE statistical model for CNV identification in targeted NGS data. Simulation analysis showed SeqCNV can detect CNVs of different sizes robustly. Additionally, we applied our method to BAC spike-in data. Compared with other methods, our method demonstrated higher sensitivity and specificity. We also tested our method on human patient datasets and causative CNVs were identified in all five samples and validated by aCGH. SeqCNV is a powerful and practical tool for CNV identification in target capture NGS data and may facilitate causative CNV discovery in genetic diseases.

Detecting CNV in targeted NGS data is a challenging area due to non-uniform distribution of read depth of target sequencing and the variation in capture efficiency. A significant difference lies in the existing tools. We think combining SeqCNV with other tools could make more reliable predictions.

A standard C++ implementation named SeqCNV can be downloaded from: http://www.iipl.fudan.edu.cn/SeqCNV.

## Additional files

Additional file 1: BAC spike-in regions. CNVs for 9 genes performed in BAC spike-in experiment. (PDF 86 kb)
Additional file 2: Additional aCGH validation. aCGH validations for copy number loss in *PRPF31* gene for other samples. (A), UTAD034; (B), UTAD069. (C), UTAD411; (D), UTAD611. (PDF 1564 kb)
Additional file 3: The criteria of control sample selection. This file describes the criteria for control sample selection. (PDF 8 kb)
Additional file 4: Execution time comparison. Estimation of the execution time for SeqCNV compared with other tools on BAC spike-in data, retinitis pigmentosa data and whole-exome sequencing data (chr1 only) in our study. (PDF 11 kb)
Additional file 5: SepCNV results on WES data. As we did on retinitis pigmentosa data, considering multiple factors such as DNA quality, DNA extraction protocol and the possibly non-even reads distribution, we randomly selected three samples to pool together as control (NA19152, NA18973, and NA19206), and randomly select one sample as case (NA10847). (PDF 7 kb)

## Abbreviations

aCGH: Array comparative genomic hybridization
adRP: Autosomal dominant retinitis pigmentosa
AIC: Akaike’s information criterion
BAC: Bacterial artificial clone
BIC: Bayesian information criterion
CBP: Candidate break point
CNV: Copy number variation
DOC: Depth of coverage
FPR: False positive rate
Indel: Insertion and deletion
MPLE: Maximum penalized likelihood estimation
NGS: Next-generation sequencing
PCA: Principal Component analysis
PEM: Paired-end mapping
PL: Penalized likelihood
RP: Retinitis pigmentosa
SNP: Single nucleotide polymorphism
SNV: Single nucleotide variant
SVD: Singular value decomposition
WES: Whole exome sequencing
