# Supplementary material for: SeqCNV: a novel method for identification of copy number variations in targeted next-generation sequencing data
Source: BMC Bioinformatics. 2017 Mar 3;18:147. doi: 10.1186/s12859-017-1566-3 (PMC5335817; doi:10.1186/s12859-017-1566-3)
Supplement: Additional file 1: — BAC spike-in regions. CNVs for 9 genes performed in BAC spike-in experiment. (PDF 86 kb) [file 12859_2017_1566_MOESM1_ESM.pdf]

Gene	Clone name	Copy number variation region
RGR	RP11-124L5	chr10:85,901,343-86,099,824
SAG	RP11-30D14	chr2:234,143,993-234,335,659
CC2D2A	RP11-79P11	chr4:15,444,618-15,622,837
TTC8	RP11-613G4	chr14:89,213,500-89,392,444
CLRN1	RP11-251C9	chr3:150,617,581-150,797,073
TULP1	RP11-78C20	chr6:35,347,479-35,521,164
LCA5	RP11-103A4	chr6:80,107,614-80,283,656
CNGA1	RP11-383J2	chr4:47,902,108-48,046,658
USH2A	RP11-360F1	chr1:216,246,056-216,442,524
