# Supplementary figures and images for: SeqCNV: a novel method for identification of copy number variations in targeted next-generation sequencing data
Source: BMC Bioinformatics. 2017 Mar 3;18:147. doi: 10.1186/s12859-017-1566-3 (PMC5335817; doi:10.1186/s12859-017-1566-3)

**A**

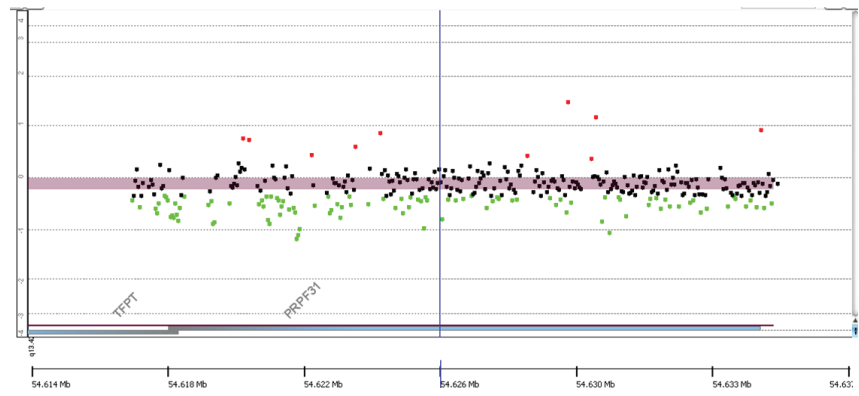

**B**

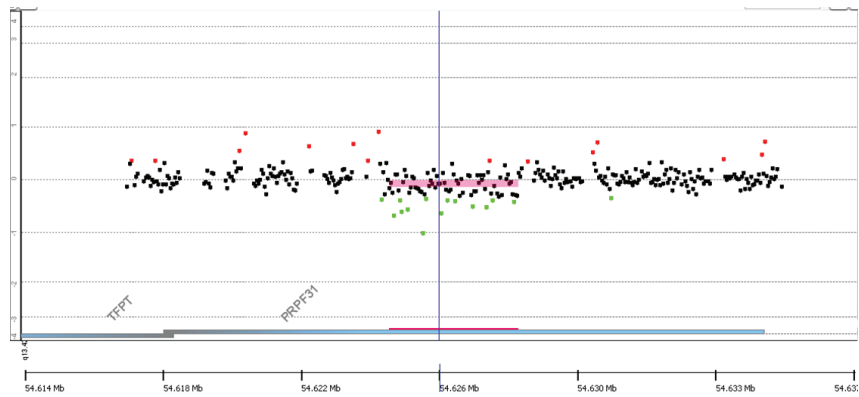

**C**

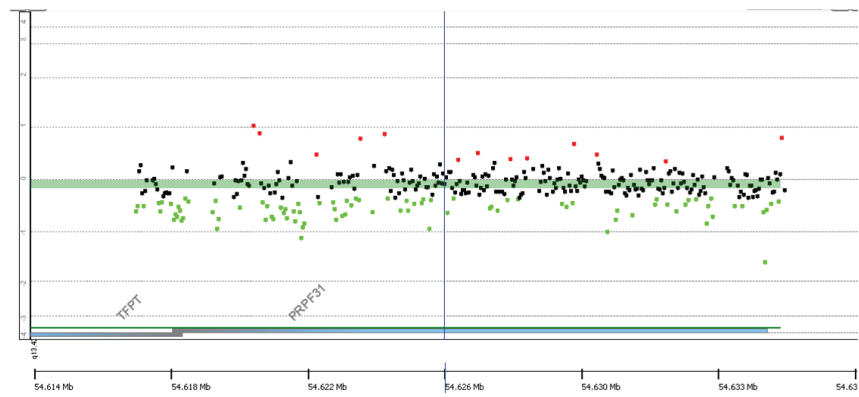

**D**

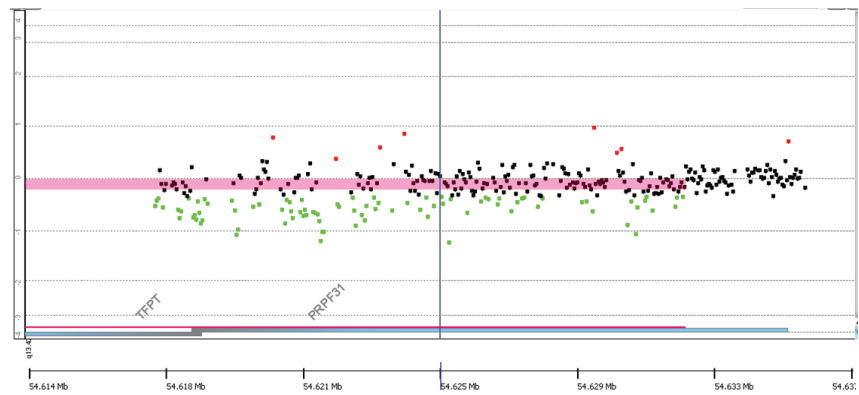

Supplement: Additional file 2: — Additional aCGH validation. aCGH validations for copy number loss in PRPF31 gene for other samples. (A), UTAD034; (B), UTAD069. (C), UTAD411; (D), UTAD611. (PDF 1564 kb) [file 12859_2017_1566_MOESM2_ESM.pdf]
