# Supplementary material for: SeqCNV: a novel method for identification of copy number variations in targeted next-generation sequencing data
Source: BMC Bioinformatics. 2017 Mar 3;18:147. doi: 10.1186/s12859-017-1566-3 (PMC5335817; doi:10.1186/s12859-017-1566-3)
Supplement: Additional file 3: — The criteria of control sample selection. This file describes the criteria for control sample selection. (PDF 8 kb) [file 12859_2017_1566_MOESM3_ESM.pdf]

Whole-exome sequencing results

All WES samples are downloaded from ftp://ftp.1000genomes.ebi.ac.uk. Like we did on retinitis pigmentosa dataset, considering multiple factors such as DNA quality, DNA extraction protocol and the possibly non-even reads distribution, we randomly selected three samples to pool together as control (NA19152, NA18973 and NA19206NA), and one sample as case (NA10847). We performed SeqCNV on chromosome 1 of these samples. To test performance of SeqCNV on WES data, we validate results of SeqCNV with the CNVs previously reported by Conrad et al. (PMID: 19812545) in the WES samples.

Control sample selection

On retinitis pigmentosa patient data, for CoNIFER and XHMM, we added extra 46 samples without copy change in *PRPF31* as control due the requirement of SVD and PCA methods. These 46 samples are normal samples and we selected them as controls for RP samples based on the following reasons:

1) Their DNA quality is good and the DNA extraction methods of these 46 and RP samples are the same.

2) The 46 normal samples and RP samples are designed to cover the same genomic regions and their number of sequencing reads are also similar.

3) The 46 normal samples and RP samples were sequenced in the same batch with RP samples.
