# Supplementary material for: SeqCNV: a novel method for identification of copy number variations in targeted next-generation sequencing data
Source: BMC Bioinformatics. 2017 Mar 3;18:147. doi: 10.1186/s12859-017-1566-3 (PMC5335817; doi:10.1186/s12859-017-1566-3)
Supplement: Additional file 4: — Execution time comparison. Estimation of the execution time for SeqCNV compared with other tools on BAC spike-in data, retinitis pigmentosa data and whole-exome sequencing data (chr1 only) in our study. (PDF 11 kb) [file 12859_2017_1566_MOESM4_ESM.pdf]

This is an estimation of the execution time for SeqCNV compared with other tools in our study. We test these CNV tools on BAC spike-in data, Retinitis Pigmentosa data and whole-exome sequencing data (chr1 only). For each tool, execution time depends on data size and capture design.

For BAC spike-in data, targeted capture design covered 3,091kbp of genome, average length of probes in design file is 200bp.

For Retinitis Pigmentosa data, it covered 25kbp of genome, average length of probes in design file is 300bp.

For whole-exome sequencing (WES), it covered 249,000kbp of chromosome 1, average length of probes in design file is 100bp.

	**SeqCNV**	**CoNIFER**	**CNVnator**	**CNVer**	**XHMM**
BAC spike-in	4 mins	9 mins	97 mins	33 mins	6 mins
Retinitis Pigmentosa	5 mins	12 mins	71 mins	26 mins	8 mins
WES<br>(chr1 only)	17 mins	58 mins	20 mins	Runtime error!	151 mins
