# Supplementary material for: SeqCNV: a novel method for identification of copy number variations in targeted next-generation sequencing data
Source: BMC Bioinformatics. 2017 Mar 3;18:147. doi: 10.1186/s12859-017-1566-3 (PMC5335817; doi:10.1186/s12859-017-1566-3)
Supplement: Additional file 5: — SepCNV results on WES data. As we did on retinitis pigmentosa data, considering multiple factors such as DNA quality, DNA extraction protocol and the possibly non-even reads distribution, we randomly selected three samples to pool together as control (NA19152, NA18973, and NA19206), and randomly select one sample as case (NA10847). (PDF 7 kb) [file 12859_2017_1566_MOESM5_ESM.pdf]

SepCNV results on whole-exome sequencing(WES) data.

All WES samples are downloaded from ftp://ftp.1000genomes.ebi.ac.uk. We use CNVs reported by Conrad et al. for validation. Like we did on Retinitis Pigmentosa data, considering multiple factors such as DNA quality, DNA extraction protocol and the possibly non-even reads distribution, we randomly selected three samples to pool together as control, and randomly select one sample as case.

control	NA19152_NA18973_NA19206
case	NA10847
precision	31%
recall	55%
false positive rate	10%
